# Supplementary material for: Attitude and preventive practices of pressure ulcers among orthopedic nurses in a tertiary hospital in Ghana
Source: PLoS One. 2023 Sep 8;18(9):e0290970. doi: 10.1371/journal.pone.0290970 (PMC10490930; doi:10.1371/journal.pone.0290970)
Supplement: Attachment — Submitted filename: REVIEWER REPORT FOR PONE-D-22-19280R2 PDF.pdf [file pone.0290970.s003.pdf]

Research Title: Attitude and preventive practices of pressure ulcers among orthopedic nurses in a tertiary hospital in Ghana

Article Identification: PONE-D-22-19280R2

Dear Editor,

Please find attached my comments on the above article which was sent to me for review.

Under the Methods

1. The first part of the first sentence "A descriptive exploratory qualitative approach" should read as "An exploratory descriptive qualitative approach"……..

2. The second sentence "The sampling technique used was purposive to analyze data using thematic content analysis" should read as "Purposive sampling approach was employed, and data was analyzed using thematic content analysis".

Under Introduction

1. The second sentence needs reference and is it annually? If this is annually, it should be indicated as such as noted at the end of the sentence "Globally, pressure injuries are the direct cause of death in 7-8% of all patients with paraplegia, with approximately 60,000 people dying of complications of pressure injuries annually (Reference?).

Under Methodology

1. The second sentence "A descriptive exploratory qualitative approach was used to obtain idiosyncratic data about the phenomenon used in this research (Elliott & Timulak 2005)" should read as "An exploratory descriptive qualitative approach was used to obtain idiosyncratic data about the phenomenon used in this research (Elliott & Timulak 2005)".

Under Sample Technique

1. Paragraph 2. Line 5-6 "This, therefore, implied that no specific sample size can be given but data was gathered until no new information was elicited". The word "elicited" as used in the sentence should be replaced with the word "forthcoming".

1. During Data Collection, how many interviews were conducted in the private room in the hospital, and how many were also conducted in participants' homes.
2. Interviews were conducted in the English language by EOA, SA, EOB, BAB. What is justification for all the four researchers conducting the interviews? How did you ensure consistency in the choice of words and sentences when four persons conducted the interviews as there was higher probability of variations in meanings of sentences, statements and words to the research participants?

Under Data Analysis

1. “DATA ANALYSIS” should be bolded and formatted in text.
2. Last paragraph, last sentence needs to be referenced “The COREQ (Consolidated Criteria for Reporting Qualitative Research) checklist consisting of 32 items was used to report the findings” (Reference). The COREQ with 32 items was developed by which researcher?

Under Analysis of Socio-Demographic Data

1. On the table, Table 1: Socio-Demographic Characteristics of Respondents, it may be important to indicate who the false names such as N1, N2, N3……N30 represent, either males or females.

2. The last word “who” as noted in the sentence should be deleted “The analysis of the result demonstrated that females dominated the gender category with a percentage of 70% as compared to males (30%). who.”

Under Negligence of patients

1. Paragraph 1, line 5, the word “professional” as stated in the sentence should read as “professionals” “This study analysis has indicated that in almost all instances, the nurses were professional in meeting all the needs of the patients to avoid negligence”. Also the sentence “This was narrated as follows” should read as “The following narratives depict what the participants said”;

Under Discussions

1. Paragraph 1, line 8-11, The sentence “This current study is supported by a study conducted by Dilie & Mengistu, (2015) stating that the lack of policies, guidelines, and lack of evidence supported by the research were factors that affected pressure ulcer practice” needs to be written as “This current study builds on a study conducted by Dilie & Mengistu, (2015) stating that the lack of policies, guidelines, and lack of evidence supported by the research were factors that affected pressure ulcer practice”.

Dr. Kwadwo Ameyaw Korsah

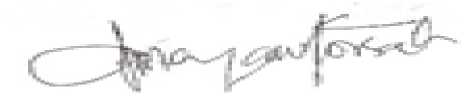

23/05/23
