# Supplementary material for: Attitude and preventive practices of pressure ulcers among orthopedic nurses in a tertiary hospital in Ghana
Source: PLoS One. 2023 Sep 8;18(9):e0290970. doi: 10.1371/journal.pone.0290970 (PMC10490930; doi:10.1371/journal.pone.0290970)
Supplement: Attachment — Submitted filename: Reviewers comment Pressure Ulcer.docx [file pone.0290970.s001.docx]

| **REVIEWER** | **COMMENT** | **RESPONSE** |
| --- | --- | --- |
|  | Reviewer #1: This is an interesting manuscript exploring nurses attitudes and preventive practices toward pressure ulcers in a hospital setting in Ghana. I acknowledge the need for studies, particularly in African countries where the incidence appears to be high with an associated burden on patients and their care providers. However, there a number of areas where this paper lacks sufficient detail for publication, which needs to be addressed.  1. The whole manuscript needs to be proofread for spelling and grammar  2. National and International guidelines for the prevention of pressure ulcers should be cited e.g. NPIAP, EPUAP, PPPIA 2019 guidelines for the prevention and treatment of pressure ulcers.  3. There appears to be no clear rationale statement, aim or research question. This should be at the end of the introduction to highlight the specific focus of the study.  4. The methods are a mix of third-person and past tense statements. A more coherent depiction of the methods and rationale are required.  5. Critical components of methodology are absent, including triangulation, reflexivity and rigour.  6. Purposeful sampling is stated, but no clear inclusion/exclusion criteria are stipulated  . 7. Some characteristics of participants appear to be redundant e.g. number of children and marital status - do you expect these to influence pressure ulcer prevention practice?  8. Themes are interesting and well documented with relevant quotes. These are interspersed with discussion points which aids the reader.  9. The discussion is a little sparse on relevant literature. 10 There is no limitations section. | Thank you  The grammatical errors has been addressed throughout the manuscript and shown in track changes  The national and international guidelines has been cited and illustrated in track changes  The aims have been included as suggested  The entire methods section has been revised  These components have been included, triangulation has been included as a limitation  Inclusion and exclusion criteria has been added  The socio-demographic section has been revised  Thanks  The entire discussion section has been reviewed  Thanks |
