# Supplementary material for: Attitude and preventive practices of pressure ulcers among orthopedic nurses in a tertiary hospital in Ghana
Source: PLoS One. 2023 Sep 8;18(9):e0290970. doi: 10.1371/journal.pone.0290970 (PMC10490930; doi:10.1371/journal.pone.0290970)
Supplement: Attachment — Submitted filename: REVIEWERS COMMENTS RESPONSE.docx [file pone.0290970.s002.docx]

| REVIEWER COMMENTS | RESPONSE |
| --- | --- |
| Reviewer #1: The rebuttal to comments to sparse and requires the reviewer to systematically check the revised manuscript. In the future, a more detailed summary of changes and the rationale for their inclusion is needed.  Substantive effort has been made to amend the document, although further additions are required. I recommend that the authors use the COREQ guidelines to ensure all key elements of the qualitative paper are included, and have a check list as a key appendix as per the GOLD standard for reporting qualitative studies.  Of the changes that have been made, the limitations section is still far too spare. The restriction to nurse only is not the only limitation of the study. More reflection is required with a full depiction in the discussion. | Thank you for taking your time to review this manuscript  COREQ SOFTWARE HAS BEEN USED TO REPORT THE STUDY AND UPLOADED AS A SUPPLEMENTARY FILE  The limitation section has been revised as shown below “The study's scope was restricted to only gathering the opinions of nurses, which may not necessarily reflect the perspectives of other important stakeholders, such as patients and physicians. As a result, future research could seek to incorporate the views of other relevant groups. Additionally, the research was limited to a single tertiary care facility, and therefore, the generalizability of the findings to other settings may be limited” |
