# Supplementary material for: Attitude and preventive practices of pressure ulcers among orthopedic nurses in a tertiary hospital in Ghana
Source: PLoS One. 2023 Sep 8;18(9):e0290970. doi: 10.1371/journal.pone.0290970 (PMC10490930; doi:10.1371/journal.pone.0290970)
Supplement: Attachment — Submitted filename: AUTHORS RESPONSE.docx [file pone.0290970.s004.docx]

| REVIEWER COMMENTS | AUTHORS RESPONSE |
| --- | --- |
| Under the Methods  1. The first part of the first sentence “A descriptive exploratory qualitative approach” should read  as “An exploratory descriptive qualitative approach”……..  2. The second sentence “The sampling technique used was purposive to analyze data using  thematic content analysis” should read as “Purposive sampling approach was employed, and data  was analyzed using thematic content analysis” | Thank you for making time out of your busy schedule to review this paper  The two sentences under the method section of the abstract have been changed as suggested and shown in the tracks. |
| Under Introduction  1. The second sentence needs reference and is it annually? If this is annually, it should be  indicated as such as noted at the end of the sentence “Globally, pressure injuries are the direct  cause of death in 7-8% of all patients with paraplegia, with approximately 60,000 people dying  of complications of pressure injuries annually (Reference?). | The sentences has been cited using Landi et al., 2007 |
| Under Methodology 1. The second sentence “A descriptive exploratory qualitative approach was used to obtain idiosyncratic data about the phenomenon used in this research (Elliott & Timulak 2005)” should read as “An exploratory descriptive qualitative approach was used to obtain idiosyncratic data about the phenomenon used in this research (Elliott & Timulak 2005)”. | The sentence mentioned has been replaced as suggested and illustrated in the track changes |
| Under Sample Technique 1. Paragraph 2. Line 5-6 “This, therefore, implied that no specific sample size can be given but data was gathered until no new information was elicited”. The word “elicited” as used in the sentence should be replaced with the word “forthcoming”.  1. During Data Collection, how many interviews were conducted in the private room in the hospital, and how many were also conducted in participants’ homes. 2. Interviews were conducted in the English language by EOA, SA, EOB, BAB. What is justification for all the four researchers conducting the interviews? How did you ensure consistency in the choice of words and sentences when four persons conducted the interviews as there was higher probability of variations in meanings of sentences, statements and words to the research participants? | Elicited has been replaced with forth coming  The information has been added as suggested see below  Four authors conducted the interviews to facilitate a comprehensive data collection process, distribute the workload, ensure inter-rater reliability, maintain consistency in data collection, and minimize potential biases. Additionally, all participants reviewed the data to address any discrepancies that arose, ensuring data accuracy and consensus among the researchers |
| Under Data Analysis 1. “DATA ANALYSIS” should be bolded and formatted in text.  2. Last paragraph, last sentence needs to be referenced “The COREQ (Consolidated Criteria for Reporting Qualitative Research) checklist consisting of 32 items was used to report the findings” (Reference). The COREQ with 32 items was developed by which researcher?  Under Analysis of Socio-Demographic Data 1. On the table, Table 1: Socio-Demographic Characteristics of Respondents, it may be important to indicate who the false names such as N1, N2, N3……N30 represent, either males or females. 2. The last word “who” as noted in the sentence should be deleted “The analysis of the result demonstrated that females dominated the gender category with a percentage of 70% as compared to males (30%). who.” | Data analysis have been boldened  Reference has been provided as suggested  This has been included as suggested  Thirty participants N1,N2,N3…..N30 (males and female nurses)  “Who”has been deleted as suggested |
| Under Negligence of patients 1. Paragraph 1, line 5, the word “professional” as stated in the sentence should read as “professionals” “This study analysis has indicated that in almost all instances, the nurses were professional in meeting all the needs of the patients to avoid negligence”. Also the sentence “This was narrated as follows” should read as “The following narratives depict what the participants said”; | The entire paragraph has been revised as followed  Negligence refers to the failure of a nurse to provide standardized care to their patients, potentially causing harm. Professional nurses are expected to consistently uphold high standards of care and avoid negligence, as it reflects negatively on their professionalism. The findings of this study consistently demonstrated that nurses were diligent in meeting all patient needs and avoiding negligence. Participants shared their experiences, highlighting the commitment of nurses to deliver professional and attentive care, thereby mitigating the risk of negligence. The following narratives depict what the participants said |
| Under Discussions 1. Paragraph 1, line 8-11, The sentence “This current study is supported by a study conducted by Dilie & Mengistu, (2015) stating that the lack of policies, guidelines, and lack of evidence supported by the research were factors that affected pressure ulcer practice” needs to be written as “This current study builds on a study conducted by Dilie & Mengistu, (2015) stating that the lack of policies, guidelines, and lack of evidence supported by the research were factors that affected pressure ulcer practice”. | The sentence has been replaced as suggested in the discussion section and shown in the tracks  Thank you |
