# Supplementary material for: Attitude and preventive practices of pressure ulcers among orthopedic nurses in a tertiary hospital in Ghana
Source: PLoS One. 2023 Sep 8;18(9):e0290970. doi: 10.1371/journal.pone.0290970 (PMC10490930; doi:10.1371/journal.pone.0290970)
Supplement: Attachment — Submitted filename: REVIEWERS COMMENT RSPONSE JULY 2023.docx [file pone.0290970.s007.docx]

| REVIEWER | COMMENTS | RESPONSE |
| --- | --- | --- |
| 1 | Under Abstract  Background  The sentence “The authors, therefore, explored the attitude and preventive practices of pressure ulcers in a tertiary hospital in Ghana” should read as “The authors, therefore, explored the attitude and preventive practices of pressure ulcers among orthopedic nurses in a tertiary hospital in Ghana” (inserted “among orthopedic nurses” as done for you. Thanks you) | Thank you for revising this statement “The sentence “The authors, therefore, explored the attitude and preventive practices of pressure ulcers in a tertiary hospital in Ghana” has been changed as suggested |
| Reviewer 1 | Under Introduction, line 3  This sentence under the introduction, line 3 in red in-text (also below) refers to which particular year or it is a projected figure or a general statement that you have made “Globally, pressure injuries were the direct cause of death in 7-8% of all patients with paraplegia, with approximately 60,000 people dying of complications of pressure injuries (Landi et al., 2007)”. Not clear, you need to indicate what I have asked you in this case. | Thank you for revising this statement “**Under Introduction, line 3**  “Globally, pressure injuries were the direct cause of death in 7-8% of all patients with paraplegia, with approximately 60,000 people dying of complications of pressure injuries (Landi et al., 2007)”. |
| Reviewer 1 | The purple part of the ensuing sentence may be considered to make the whole sentence stand out clearly “The first part (section A) consisted of the socio-demographic data of the participants which was intended to establish rapport with the research participants in order to get rich data in the subsequent sections. The other sections were based on the study objectives.  (I have inserted it for you as appeared above in-text “which was intended to establish rapport with the research participants in order to get rich data in the subsequent sections” Please you may this change as suggested above. Thank you).  The sentence in parenthesis in purple colour below is noted before paragraph 3 under the **Under Sampling Technique** “The questions had probes to support the main questions”.  Note that probes are not supposed to be part of the original interview questions. Probes are follow up questions during the interview and so are not supposed to be part of the interview questions. However, as the research participant speaks, or during the course of interview conversation, follow up questions may be asked as probes.  In view of this above explanation, the sentence “The questions had probes to support the main questions” may be written as “During interviews in this study, probes were asked for clarifications of issues which were raised”.  **Under Sampling Technique**  What informed your decision to conduct this research at the Korle-Bu Teaching Hospital and not in any other hospital in Ghana? Indicate this in paragraph 3, line 4, where you have mentioned the Korle Bu Teaching Hospital. (Where I have highlighted Korle-Bu Teaching Hospital in red ink in-text. Thanks)  **Under Sampling Technique**  Paragraph 4, lines 5 – 6, you have made the following sentence “Additionally, all participants reviewed the data to address any discrepancies that arose, ensuring data accuracy and consensus among the researchers”. – How did you do this? Can you expand on how you went about this by explaining further? | Thank you for making these changes. Changes accepted  Changes accepted as suggested. Thank you  Thak you changes suggested have been effected  Changes have been effected in the manuscript as you suggested  The reason for choosing KBTH have been added  That statement has been deleted.  Thanks |
| Reviewer 1 | **UNDER RESULTS**  **Analysis of Socio-Demographic Data**  Please, you were asked to indicate on the table 1: Socio-Demographic Characteristics of Respondents, the sex of each research participant. For instance N1 (male or female), N2 (female or male), N3 (male or female), N4 (female or male) N5………………..N30.  It is important that you have indicated the overall number of males (9) and females (21) as found on the table 1 (I have highlighted in red ink on the table). However, it may imperative to show individual sexes on the table 1 to give sense of who is that particular participant, especially as you have used the N1, N2, N3, N4, N5……….N30 as pseudonyms in the findings section.  **UNDER RESULTS**  **Under Busy schedules and lack of time**  **Paragraph 1, line 4, the word “However” should start small letter and read as “however”.** | The participants’ genders have been included as suggested  A small letter has been used to replaced the capitalize letter of the “H” as suggested. Thank you |
| Reviewer 1 | Under Discussions  **Paragraph 5, last sentence,** facilitate healing(Boyko, Longaker, & Yang,2018). Please bring the space between healing and the reference (Boyko, Longaker, & Yang,2018). It has been highlighted in-text with red ink. | Thank you  The spacing has been corrected as suggested |
|  |  |  |
|  |  |  |
