# Supplementary material for: Attitude and preventive practices of pressure ulcers among orthopedic nurses in a tertiary hospital in Ghana
Source: PLoS One. 2023 Sep 8;18(9):e0290970. doi: 10.1371/journal.pone.0290970 (PMC10490930; doi:10.1371/journal.pone.0290970)
Supplement: Attachment — Submitted filename: REVIEWER REPORT .doc [file pone.0290970.s008.doc]

**REVIEWER’S REPORT**

**PONE-D-22-19280R4**

**Attitude and preventive practices of pressure ulcers among orthopedic nurses in a tertiary hospital in Ghana**

**UNDER RESULTS** Analysis of Socio-Demographic Data

Please, you were asked to indicate on the table 1: Socio-Demographic Characteristics of Respondents, the gender or sex of each research participant. For instance N1 (male or female), N2 (female or male), N3 (male or female), N4 (female or male) N5………………..N30. It is important that you have indicated the overall number of males (9) and females (21) as found on the table 1. In the current submission, I have observed that the gender or sex of the research participants were not indicated on the table as suggested, however the gender or sex of most of the participants were indicated at the end of the quotes but not all of them, some inconsistencies are here. If you like to keep them as they are now, you may have to indicate at the end of each quote the sex or the gender of the research participants.

I am satisfied about the responses to the queries which raised in other sections of the article.

I suggest that it may be accepted for publication.

Thank you

Dr. Kwadwo Ameyaw Korsah
